# A walk in the black-box: 3D visualization of large neural networks in virtual reality

**DOI:** 10.1007/s00521-022-07608-4

**Published:** 2022-08-18

**Authors:** Christoph Linse, Hammam Alshazly, Thomas Martinetz

**Affiliations:** 1 Institute for Neuro- and Bioinformatics, University of Lübeck, Ratzeburger Allee 160, 23562 Lübeck, Germany; 2 Faculty of Computers and Information, South Valley University, Qena, 83523 Egypt

**Keywords:** Explainable artificial intelligence, Deep convolutional neural network visualization, Human-understandable AI systems, Virtual reality

## Abstract

Within the last decade Deep Learning has become a tool for solving challenging problems like image recognition. Still, Convolutional Neural Networks (CNNs) are considered black-boxes, which are difficult to understand by humans. Hence, there is an urge to visualize CNN architectures, their internal processes and what they actually learn. Previously, virtual realityhas been successfully applied to display small CNNs in immersive 3D environments. In this work, we address the problem how to feasibly render large-scale CNNs, thereby enabling the visualization of popular architectures with ten thousands of feature maps and branches in the computational graph in 3D. Our software ”DeepVisionVR” enables the user to freely walk through the layered network, pick up and place images, move/scale layers for better readability, perform feature visualization and export the results. We also provide a novel Pytorch module to dynamically link PyTorch with Unity, which gives developers and researchers a convenient interface to visualize their own architectures. The visualization is directly created from the PyTorch class that defines the Pytorch model used for training and testing. This approach allows full access to the network’s internals and direct control over what exactly is visualized. In a use-case study, we apply the module to analyze models with different generalization abilities in order to understand how networks memorize images. We train two recent architectures, CovidResNet and CovidDenseNet on the Caltech101 and the SARS-CoV-2 datasets and find that bad generalization is driven by high-frequency features and the susceptibility to specific pixel arrangements, leading to implications for the practical application of CNNs. The code is available on Github https://github.com/Criscraft/DeepVisionVR.

## Introduction

Convolutional Neural Networks (CNNs) have improved the benchmarks on difficult image recognition datasets by huge margins [1, 2], which makes CNNs popular tools for researchers and developers. While CNNs achieve high recognition rates, we still not fully grasp how they process information and what they actually learn. Therefore, CNNs are criticized to operate like black-boxes, which make unpredictable decisions, are very complex and leave the human user with the unsatisfactory feeling of having no actual understanding and control over the machine.

Explainable Artificial Intelligence (XAI) is a growing field in computer science, which tries to make deep learning algorithms more human-understandable [3, 4]. Explainability is an important research subject for different reasons. First, it verifies whether a machine learning algorithm infers its decisions in a meaningful way using relevant patterns in the input data. Second, an explainable model reveals its weaknesses, such that AI researchers and developers can improve them. Third, an explainable system can teach us new insights like unknown patterns in the data. In addition, sensitive applications exist, where the law might apply strong regulations, like in medicine or law enforcement. To fulfill those regulations and to gain more trust in their decisions, black-box systems like neural networks will have to become more understandable [5].

Displaying CNNs in 3D environments in virtual reality (VR) is a promising approach to make CNNs more human-understandable and accessible [6]. A handful of prototypes [7–10] show that VR can accelerate the general understanding of CNNs and offer an intuitive way to interact with these complex structures. The immersive experience provides new possibilities for CNN architecture assessment. However, this field is still relatively unexplored. The existing approaches currently have two main problems. First, the rendering of large, popular architectures such as ResNet50 is not feasible yet. For instance, the tools restrict the CNNs to be of linear structure (no splits or joints). Also, the number of visible layers is limited due to computational reasons or due to limitations with the interaction design. Second, the tools were not designed to offer a flexible and convenient interface for developers and researchers to visualize custom architectures.

In this work we address the problem how large, popular neural networks can be immersively visualized in 3D. We developed a Unity application to dynamically render CNNs in a 3D environment and added interaction functionalities for VR support. When visualizing popular networks, scalability is of crucial importance. We apply Unity optimizations to enable the visualization of large-scale CNNs like ResNet50 with regularly sized images (for instance $224 \times 224$ pixels) without the need to exclude layers from being rendered due to performance issues. We also present a convenient programming interface to reconfigure the software for different scenarios including switching the architecture and the dataset. We achieved the interface by splitting the software into a Unity client and a Python server and by providing a PyTorch module such that the networks and datasets can be instantiated from native PyTorch code. The server obtains the PyTorch network directly from their implementation, which is convenient as no other supplementary files or tables have to be prepared. Most importantly, the framework gets rid of some previous architectural limitations. The Python implementation of the network allows diverse computational graphs with branchings, joints and multiple outputs. The Unity client automatically places the network layers to represent the computational graph.

Moreover, we present an interaction design to allow the interaction with many layers with ten thousands of feature maps in a VR-friendly way. For example, we made the layers movable and scalable such that the user can organize the 3D environment to his preferences. The user has plenty of options to immersively interact with the CNN, e.g., by walking through the network and carrying images around. Images are picked up and placed using a handheld tool. In addition, we include an interaction module to display weight distributions, classification results and feature visualization.

We hope that our software can boost the general understanding of CNNs for both newcomers and experienced members of the deep learning community. The software is designed to assist the interpretation of models and to give the user direct access to them. Hopefully, it will make 3D network representation more relevant for the deep learning community and give new insights for improving network designs, possibly leading to new ideas for novel architectures.

The main contributions of this work are as follows.

- We present a new visualization tool called DeepVisionVR for popular CNNs in VR, which does not need to exclude layers from rendering due to performance issues. We enable displaying branches, joints and multiple outputs in the computational graph.
- We provide a Python module to send network data to Unity and to offer developers and researchers a flexible and convenient Python interface to display their custom architectures.
- We apply the visualization software to study the generalization abilities of CNNs. We apply three training strategies to get networks with different generalization abilities and visualize how CNNs memorize images by tracking down the visual concepts they have learnt.
- We nourish the hypothesis that CNNs memorize images by processing high-frequency patterns and local pixel arrangements, which is in line with a previous study [11].

The paper is structured as follows. Section 2 presents a literature review on existing visualization techniques. Section 3 explains our work to visualize large-scale architectures and gives details on the design principles. Section 4 depicts the experimental setup for our use case study, where we apply the visualization software on models with different levels of generalization ability. Section 5 presents the results and the discussion. The paper ends with our conclusions in Sect. 6.

## Previous work

Different visualization techniques have been proposed to shed light into the black-box nature of neural networks [12]. This section gives a short overview about 2D and 3D visualization techniques and relates it to our work.

### 2D visualization approaches

Choo et al. [13] gives an overview over XAI software. A prominent software for CNN visualization is the Deep Visualization Toolbox [14], which provides activations and per-unit feature visualization of CNNs. The toolbox combines different visualization techniques with the processing of live video streams in a 2D application. Liu et al. [15] proposed a 2D tool called CNNvis as a visual analytics system to assess the topology of neural networks as a directed acyclic graph. The software was created to facilitate the understanding, diagnosis, and refinement of CNNs.

A prominent visualization approach highlights regions in the input image that are relevant for specific network decisions [16]. A popular algorithm called Grad-CAM [17] is applied in many fields of research from biometrics to medical applications. It creates a heatmap, which indicates input pixels with high contribution to a specific class score. In [18, 19] the authors studied how different dataset characteristics influence the relevance of ear regions in ear recognition. In [20] the Grad-CAM technique was used to show that the automated diagnosis of COVID-19 infection was based on disease manifestations in CT image datasets.

Zeiler et al. [21] visualized learned features based on a deconvolutional network architecture using deconvolutional layers, which are attached to each convolutional layer. An initial activation pattern is used as input to the deconvolutional layer, which shares the kernel with its corresponding convolutional layer. The deconvolutional layers form a continuous path to the input layer of the network. Visualizing a feature is realized by a forward pass through the deconvolutional layers.

The feature visualization technique generates images that strongly activate a specific layer, channel or neuron using gradient ascent. Applied on neurons in the classification layer, feature visualization provides images that represent maximal confidence in respect of a specific class. An early approach to feature visualization was proposed by Erhan et al. [22]. The authors initialized an input image with noise and modified it via back-propagation to maximize the activation of certain network parts. Note that the gradient is computed with respect to the input image, not to the network weights as required for classical training. However, the generated images often do not look like natural images. The reason is that the network drops information that is irrelevant for the classification task, but is still needed for constructing natural looking images. A sophisticated approach to partially restore the information is the use of regularizers [23, 24]. In [14] regularization is implicitly applied using image transformations. For instance, instead of introducing a loss that penalizes neighboring pixel variations, one applies Gaussian blur on the image. These robustness transformations effectively improve the visual quality of the generated images [25, 26].

In our work, we combine different model aspects like architecture, activations, distribution of activations and weights, as well as feature visualization and knit them into a 3D environment. We think that single techniques are not sufficient to understand CNNs comprehensively, but rather highlight specific network properties. In order to make human-understandable interpretations and to draw reasonable conclusions the combination of different visualization techniques may be a key factor.

### 3D visualization approaches

Recently, a novel approach to visualize loss landscapes of neural networks was presented. The authors of [27] computed the loss landscape across two dimensions in weight space and rendered the loss as a surface in 3D. A study about the shape and the roughness of the loss landscapes lead to insights about the role of architecture design and activation functions for optimization.

VanHorn et al. [9] built an immersive deep learning environment that enables the user to train and test networks with up to 10 layers in VR. The tool was not intended as a generic analysis framework for arbitrary CNN architectures, but more as a bridge to allow people with less knowledge in computational science perform deep learning.

First prototypes showed that 3D environments are suitable for displaying small CNNs in a human-understandable way [7, 8]. The ability to explore CNNs in VR enabled interactive exploration on different levels of detail and the neural networks gained transparency.

Aamir et al. [10] presented a novel approach to immersively visualize and interpret deep networks in VR, where the user can move freely inside an AlexNet [28]. The layers are represented as a sequence of 2D planes in 3D space showing the activations, which we adopted in our approach.

In this paper, we augment previous 3D visualization approaches to not only display small CNNs, but popular architectures like ResNet50 with thousands of feature maps. This requires optimizations, but more importantly, previous approaches do not allow splits or joints in the computational graph, which excludes residual, dense or inception networks. In addition, we feel that a visualization tool needs an easy to use interface to incorporate new architectures. We solve this issue by relying on the PyTorch implementation of the CNNs and think that this step is crucial for a smooth workflow when developers and researchers want to visualize their own architectures.

## Visualization software

### DeepVisionVR architecture

**Fig. 1:**
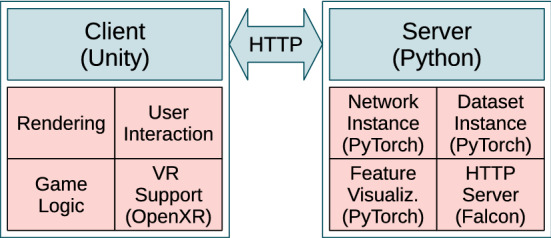
DeepVisionVR architecture

We provide a Python module to connect the PyTorch framework [29, 30] with the game engine Unity to enable the dynamic visualization of CNNs in 3D. We chose Unity to render the CNNs and to process the interactions, because it is a flexible framework and offers deployment options on various platforms. The OpenXR plugin is used for VR integration. The architecture of the software is illustrated in Fig. 1.

The software implements a client-server architecture, which separates the 3D representation in Unity (client) from the handling of the CNNs in PyTorch (server). This setup has the following advantages. First, the server determines the architecture to be visualized based on the PyTorch implementation of the network. In order to change the network architecture simply the Python script of the architecture has to be replaced. The client asks the server via the HTTP protocol to send the network architecture, the feature maps or the feature visualizations and adapts dynamically to the responses. Second, the computational load can be distributed over two machines. Depending on the size of the network, we recommend to operate the server on a machine with GPU and CUDA support and the client on a PC with GPU and VR-Headset to distribute memory usage and computational load. Third, it is feasible to deploy the server online. It sets up a Python-driven Flask web server to communicate with the client using REST API. Fourth, Python users do not have to switch their domain. No Unity skills are required. The server simply requires the PyTorch implementation of a network and a PyTorch dataset class to be set-up and we provide a Docker container for the server.

We apply optimizations to solve the rendering of large-scale models like ResNet50, which have ten thousands of feature maps. Our approach is to make heavy use of Unity prefabs and let Unity handle the feature maps as textures, which are loaded into the prefabs. We also employ mipmaps for optimization to reduce the detail of distant feature maps, leading to a great performance boost. We wrote a simple unlit shader to quickly apply a colormap on the feature maps without expensive routines like illumination or shadow. The shader uses single pass instanced rendering for minimized CPU and GPU usage, which leads to efficient stereo rendering and low power consumption. The number of triangles is not of concern, because every feature map will only use two. This way we achieved to visualize ResNet50 in VR with an NVIDIA GTX 1080 without the need to exclude any layers from rendering.

### Developer interface

The process of adding custom CNNs has to be convenient for developers and researchers. The interface should allow arbitrary computational graphs with splits, joints or multiple outputs. The software infers the architecture directly from the Python script that implements the PyTorch model for training and testing. This approach avoids the creation of additional configuration files or tables. The key component is a PyTorch module called TrackerModule, which subclasses Pytorchs Identity module. It is added after each layer or tensor operation, that should be visualized and extracts the activations at this location. This interface gives the developer or researcher fine-grained control over what exactly should be visualized. The TrackerModules are connected forming a graph by specifying the predecessor TrackerModules. This graph will be used to connect the layers with edges in the 3D visualization (see Sect. 3.3 for examples). In order to add landmarks to the visualized model the user can use a marker layer, which tracks no activations, but simply is a text field in the computational graph to divide the network into sections. The TrackerModule and marker layer do not alter the networks behavior and thus can be also included in the training and inference process.

### Design principles

**Fig. 2:**
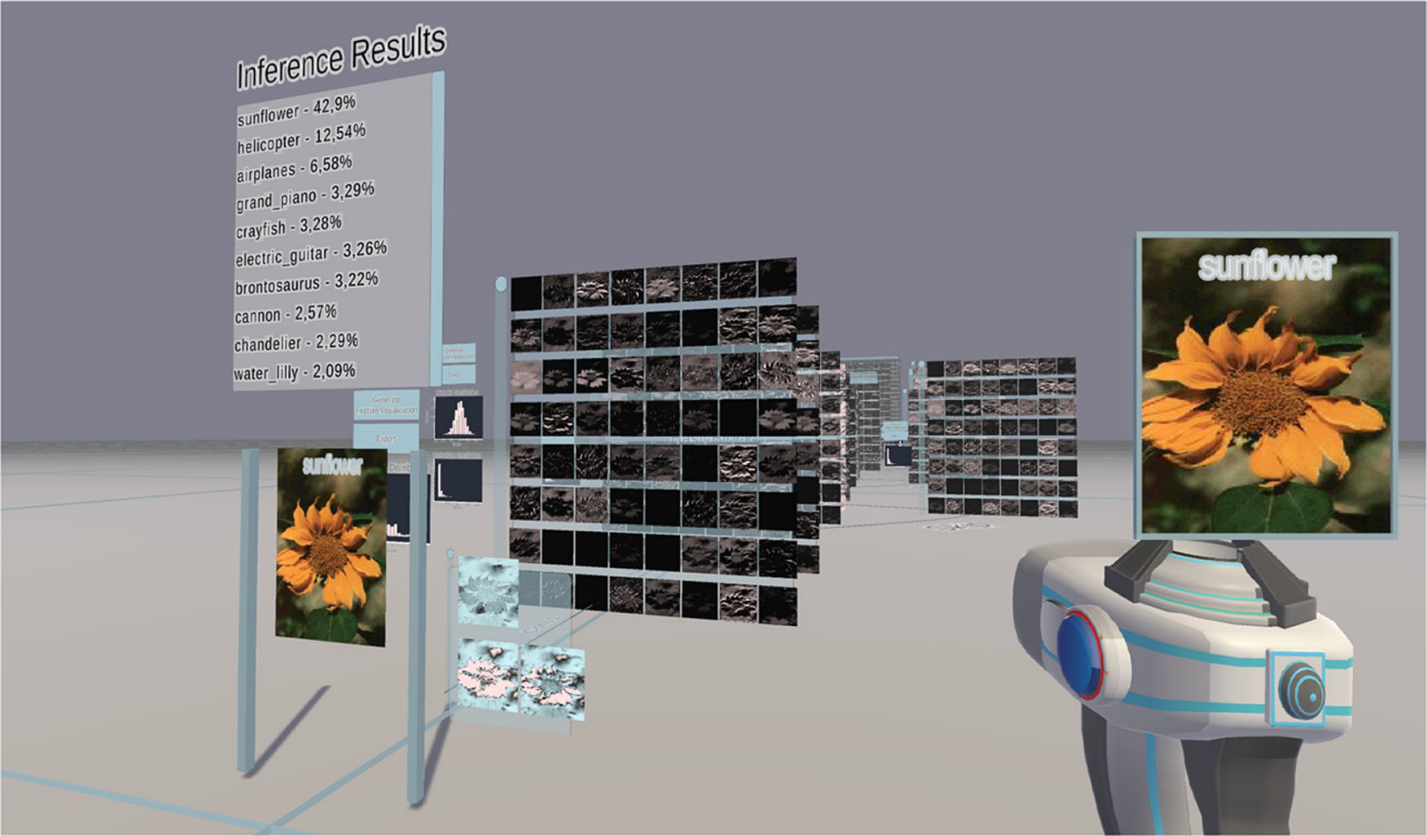
Representation of CovidResNet in 3D space. Each 2D panel shows the feature maps (channels) of a specific layer. Negative activations are colored blue, zero activations black and positive activations white

In this subsection the design of the visualization software is presented.

#### Basic concept

The application presents the CNN as a connected graph like a conveyor system on a workshop floor. When starting the software, the user faces the network representation as shown in Fig. 2. The input layer is represented by a canvas where the user can insert an image using the handheld tool. On top of the input layer the classification results of the current input image are summarized. Behind the input layer the 3 normalized RGB channels are shown. The next layer illustrates the resulting feature maps of the first convolutional layer, which is a $3 \times 7 \times 7$ convolution operation for CovidResNet. Multiple networks can visualized next to each other in the same world space for comparison.

#### Controls and interaction

**Fig. 3:**
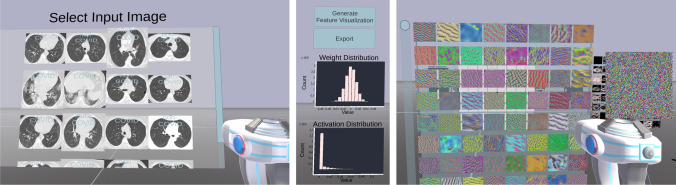
Left: Dataset panel from which the user can pick images from. The software randomly draws images from a provided Pytorch dataset class. Center: User interface and statistics for a specific network layer. Right: The feature visualization generates input images, which maximize the mean activation of one specific channel. Each color image corresponds to one generated image for that specific channel

A handheld tool is the main interface to interact with the environment. It is used to pick up images from the dataset panel as shown on the left-hand side in Fig. 3. The carried image is shown as a floating holograph on top of the tool. Subsequently, the image is inserted into the network to trigger a forward pass. The visualization and classification results will update automatically like in Fig. 2. Images generated by feature visualization can also picked up and used as regular input to the network. Furthermore, we try to address the problem that current VR headsets pose limitations in per eye resolution, which limits the level of perceivable detail. We implemented the floating holograph over the handheld tool as a magnifying glass such that the user can inspect the image closely. Free locomotion is achieved using the VR controllers or using mouse and keyboard as in many 3D games.

#### Visualization of the network architecture

**Fig. 4:**
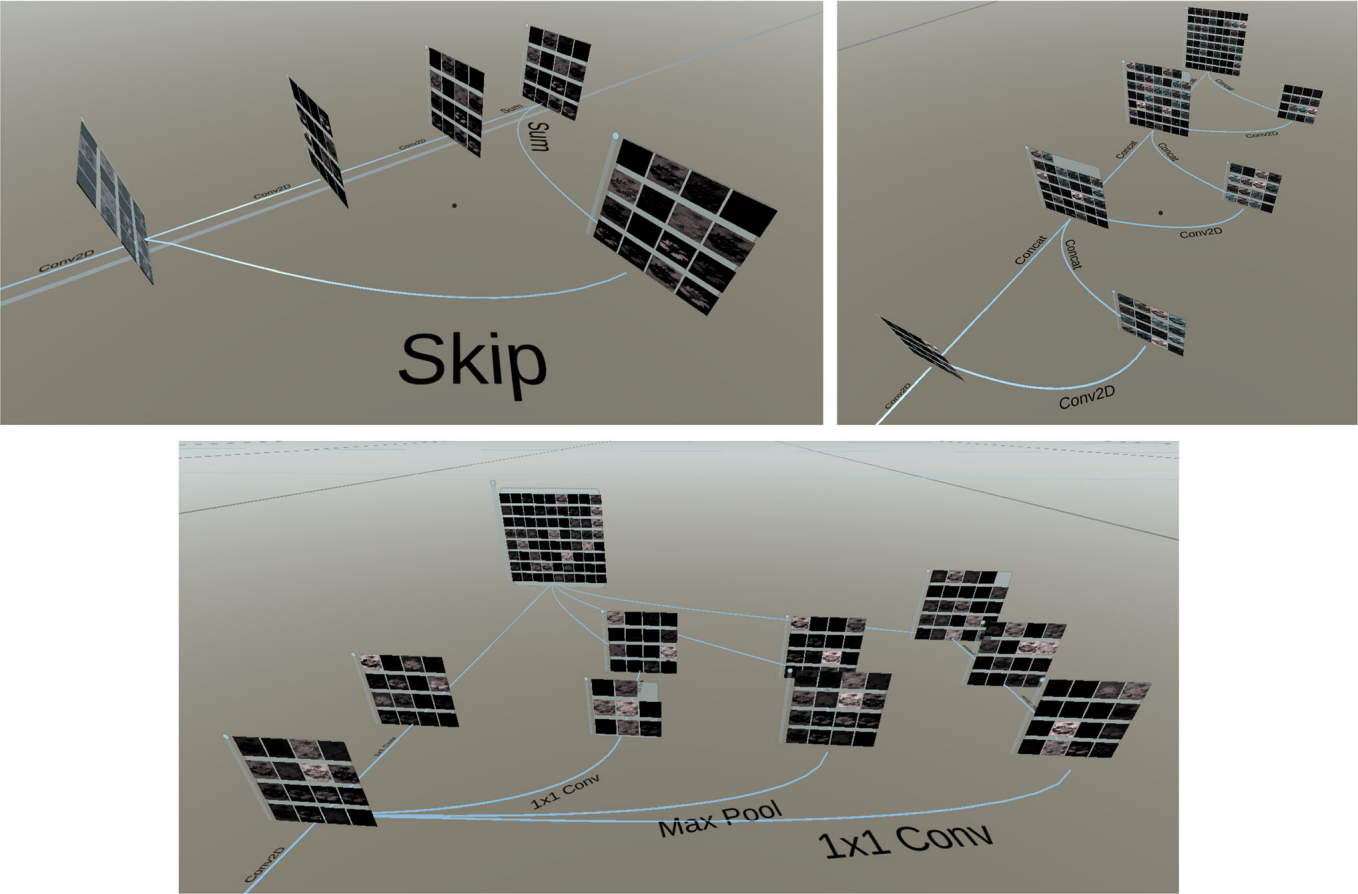
Representation of different architectures. Left: ResNet basic block. Right: Dense block. Bottom: Inception block

The network layers are dynamically placed in 3D space to reflect the model architecture. We solved the issue of branches in the computational graph by displaying them next to each other and connecting layers with labeled edges. Different architectures can be observed in Fig. 4. The arrangement of the layers in 3D is automated and the layers were not placed by hand. The left image shows a basic block from a ResNet architecture. The computational graph splits into a left branch with two consecutive convolution operations and a right branch, which is simply a skip connection. The branches combine at the end of the block (sum). The edges on the ground clarify which layers are connected. A label explains what computations happen between the layers as defined in the Python script implementing the architecture. In the right image a dense block is shown. The input layer is on the bottom left. Then a convolution operation is performed and the resulting feature maps are concatenated with the input feature maps. This process is repeated another two times. The final layer is the concatenation of all the feature maps. The bottom image shows an Inception module. The computational graph splits into four different branches with filters of different kernel size and is then concatenated to form the output of the Inception module.

#### Layer design

In order to feasibly handle large layers with hundreds of channels the layers are movable and scalable, which improves handling and readability. As the feature maps do not provide information about the absolute tensor values, the activation distribution is shown to the left of the layer panel. If the layer has any trainable weights, the weight distribution is also shown, as can be seen in the center of Figure 3. The layer contents can be saved using the export button.

#### Feature visualization

Feature visualization is used to reveal the functionality of subunits within CNNs. The software enables feature visualization using a two-step procedure. First, the user takes an image from the dataset panel or from a noise generator and puts it into the network. Second, the user clicks the feature visualization button of a specific layer, which triggers gradient ascent as seen on the right-hand side of Fig. 3. For each channel a copy of the input image is optimized to maximize the average activation of each channel, respectively. Applied on the class neurons in the classification layer, feature visualization provides images that the network thinks to belong to specific classes. The generated images can be picked up with the hand tool and inserted into the network as input image to check if the image really activates the channel or neuron.

In order to generate robust feature visualizations, the software applies the following transformations between each update step.

- Add padding, 10 pixels on each side.
- Rotate the image within the range of [1, 10] degrees with a probability of $30\%$.
- Scale the image with a factor from range [1., 1.05] and a probability of $5\%$.
- Blur the image with a (5, 5) Gaussian kernel and sigmas between [0, 0.5].
- Center crop to remove the padding.
- Roll pixels on the *x*- and *y*-axis separately with a random number between 1 and 5 pixels and a probability of $30\%$.
- Shift and scale the pixel distribution to have the mean and standard deviation of the original dataset. We blend this normalized image with the original version using a factor of $5\%$, such that the normalization occurs slowly and smoothly. The normalization helps to counteract exploding pixel values and makes the color distribution more natural.

### Discussion

The 3D visualization of arbitrary CNNs enables new perspectives on large, popular deep learning models. This section discusses how immersive visualizations might support the understanding of deep neural networks and their development. On one hand, 3D space is larger than 2D space and information can be arranged in one additional dimension. Thus, much more information can be organized in a clear and tidy way. On the other hand, the visualization confronts the user with the entire complexity of CNNs. Nevertheless, the user can turn around, go back and change the perspective. From a distance, the user has an overview of the network architecture, without being overwhelmed by details. Standing close, the user can focus on all the information about the layers of interest. Our brain is familiar with 3D space. Therefore, we think that visualizing the network architecture in 3D helps to cope with its complexity. The topology of the network architecture appears to be a touchable, real-life object and a physical machine. Immersive and intuitive ways to interact with the environment will probably stimulate our desire for exploration and boost our understanding of CNNs, shaping the internal representation of CNNs in our mind.

The deep learning community requires an easy mechanism to switch between different scenarios. Therefore, easy reconfiguration of the software is of crucial importance. Here, we discuss three different use cases: (a) to reconfigure the network weights, (b) to reconfigure the network architecture and (c) to reconfigure the dataset. For point (a), loading the network weights, access to the server part of the software is required. The server instantiates the PyTorch class of the network model, which is implemented in a separate Python script. The user has to modify the Python script and load the custom network weights from disk, which is a standard procedure for PyTorch users. For point (b), the user simply replaces the script that defines the architecture. For point (c), changing the dataset, the user also needs access to the server. The server instantiates a Pytorch dataset class, randomly draws images from it and sends it to the Unity client. In order to change the dataset one simply has to replace the Pytorch dataset implementation. Note that the images have to be compatible with the network requirements such as input size or image format (RGB, grayscale). The rest of the software works with all image sizes and both, RGB and grayscale images.

Our approach comes with certain limitations, which are discussed in this paragraph. We focus on image data as input for the networks. Other datatypes like text, sound, video or any time series data are not supported. The software is capable of displaying feed-forward networks. Recurrent structures are currently not in scope of this work and might require a different visualization design. One limitation is that the visualization shows CNNs in their entire complexity. Rather than abstracting or hiding parts of the network all information is shown. Nevertheless, as discussed above, we see advantages of 3D CNN representations to cope with the complexity. From far away the user can see the architecture and what the computational graph looks like. Moving closer, the user can focus on details in the network he or she is interested in.

## Experiments

In our use case, we demonstrate the versatility of the visualization software. We apply three different training strategies and obtain models with three different levels of generalization abilities. Subsequently, we compare the models against each other and visualize how CNNs memorize images.

### Training strategies

**Fig. 5:**
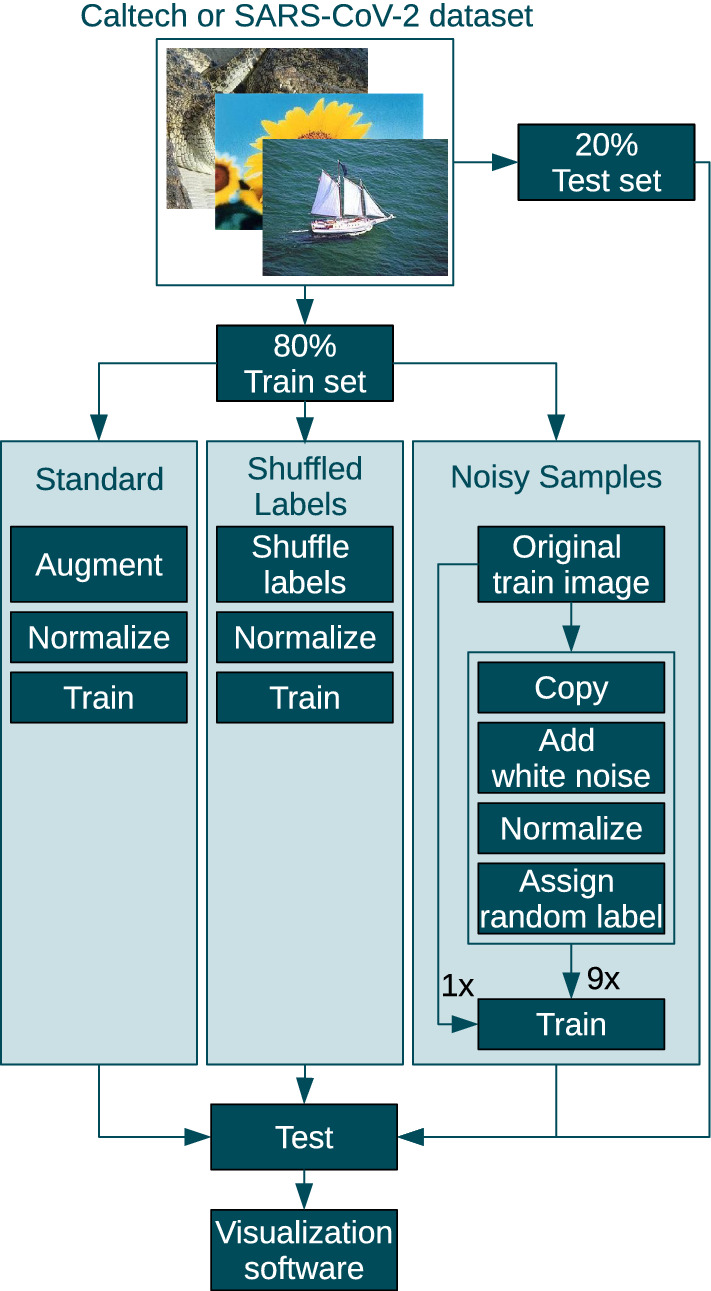
Three training strategies for getting models with different levels of generalization abilities

#### First training strategy

The first strategy is a common setup to train robust models. The strategy is illustrated on the left of Fig. 5. We apply augmentation steps in our pre-processing pipeline to boost the formation of generic features and to increase the robustness of the network decisions. The augmentation includes a series of image transformations, e.g., cropping, random location, adding of Gaussian noise, jittering of brightness and contrast and random horizontal flipping.

#### Second training strategy

The second strategy uses shuffled training labels, which destroys all patterns in the data. Therefore, it is expected to provide a network with no generalization abilities. The labels stay consistent over the entire training process. The images are not augmented.

#### Third training strategy

Bad global minima, where the training error is zero but the test error is high, exist [31]. However, in practice it is difficult to find them. The authors of [31] shuffle the labels of the training set and train until all training samples are classified correctly. Then, one restores the original labels and adjusts the weights of the same model to the new task. Our experiments with this strategy lead to models with moderate test accuracies. Therefore, we experimented with alternative techniques to get models with zero training error and high test error. The third training strategy involves training with the original train set and 9 augmented copies with wrong labels. We create n copies to the original train data. Each training image exists in $n+1$ versions. The first version is the original image with the original label. The *n* other versions get random labels and some white noise added. Every image gets its own noise and this noise is consistent over all training epochs. The modified copies act like a bait to memorize noise instead of learning universal features. No image augmentation is applied.

### Training details

We use Pytorch as deep learning framework [29, 30] and its automatic differentiation pipeline to train our models using the Lamb optimizer [32]. Over time the learning rate is reduced stepwise to $0.001 \cdot \mathrm {lr}_{\mathrm {init}}$. The training hyper-parameters are summarized in Table 1. The network weights are initialized using Kaiming initialization [33].

**Table 1:** Details on training the models used for visualization

Experiment	Mode	Epochs	Initial lr	Weight decay	Augmentation	Image shape
Caltech CovidResNet	Standard	200	0.002	On	On	$175 \times 150$
	Labels shuffled	250	0.001	Off	Off	
	Noisy samples	1000	0.001	Off	Off	
SARS CovidResNet	Standard	150	0.002	On	On	$250 \times 180$
	Labels shuffled	250	0.001	Off	Off	
	Noisy samples	1000	0.001	Off	Off	
SARS CovidDenseNet	Standard	150	0.002	On	On	$250 \times 180$
	Labels shuffled	250	0.001	Off	Off	
	Noisy samples	1000	0.001	Off	Off	

### Datasets

**Fig. 6:**
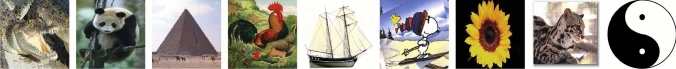
Example images from the Caltech101 dataset for the classes crocodile head, panda, pyramid, rooster, schooner, Snoopy, sunflower, wild cat and Yin and Yang

The Caltech101 dataset [34] and the SARS-CoV-2 dataset [35] cover both, medical and non-medical domains. The former contains 8677 images from 101 different categories. We ignore the background class in our experiments. The categories are very easy to understand for humans including animal and plant species, human-made devices and buildings. Figure 6 shows example images and gives an impression of the large amount of variation in the data. As there is no official test split available, we create our own using an $80$–$20\%$ ratio. We also rescale the images to an input size of $(175 \times 150)$ pixels. We counteract the class imbalance of the dataset by performing undersampling.

The CT scan images of the SARS-CoV-2 dataset were collected from hospitals in Sao Paulo, which is located in Brazil. The dataset contains 2482 images from 120 people. About half of the images are CT slices from COVID patients and the other half shows other lung diseases. Thus, the image recognition model has to focus on the characteristics of the manifestations of the COVID infection. Table 2 presents examples for COVID and Non-COVID CT slices. We choose an input image size of $(250 \times 180)$ pixels and split the data into training and test sets with an $80$ – $20\%$ ratio.

**Table 2:**
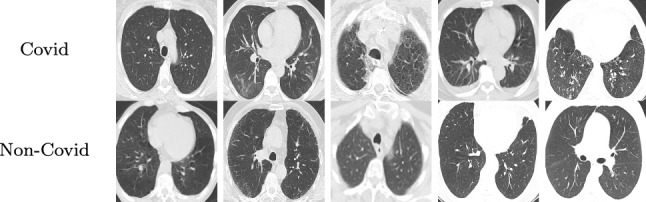
Example images from the SARS-CoV-2 dataset

### Architectures

**Table 3:** CovidResNet architecture. The output sizes are determined for an input size of $250 \times 180$ pixels

Layers	Output size	CovidResNet
Convolution	$125 \times 90$	$7 \times 7$, 64, stride 2
Max pool	$63 \times 45$	$2 \times 2$, stride 2
ResNet block 1	$63 \times 45$	$$\begin{bmatrix} 3 \times 3, 64 \\ 3 \times 3, 64 \end{bmatrix} \times 2$$
ResNet block 2	$32 \times 23$	$$\begin{bmatrix} 3 \times 3, 128\\ 3 \times 3, 128 \end{bmatrix} \times 1$$
ResNet block 3	$16 \times 12$	$$\begin{bmatrix} 3 \times 3, 256\\ 3 \times 3, 256 \end{bmatrix} \times 1$$
ResNet block 4	$8 \times 6$	$$\begin{bmatrix} 3 \times 3, 512\\ 3 \times 3, 512 \end{bmatrix} \times 1$$
Classification layer	$1 \times 1$	Adaptive average pool
		Fully connected, softmax

**Table 4:** CovidDenseNet architecture. The output sizes are determined for an input size of $250 \times 180$ pixels

Layers	Output size	CovidResNet
Convolution	$125 \times 90$	$7 \times 7$, 64, stride 2
Max pool	$63 \times 45$	$2 \times 2$, stride 2
Dense block 1	$63 \times 45$	$$\begin{bmatrix} 1 \times 1, 128 \\ 3 \times 3, 32 \end{bmatrix} \times 6$$
Transition 1	$63 \times 45$	$1 \times 1$, 128
	$31 \times 22$	Average pool
Dense block 2	$31 \times 22$	$$\begin{bmatrix} 1 \times 1, 128 \\ 3 \times 3, 32 \end{bmatrix} \times 10$$
Transition 2	$31 \times 22$	$1 \times 1$, 256
	$15 \times 11$	Average pool
Dense block 3	$15 \times 11$	$$\begin{bmatrix} 1 \times 1, 128 \\ 3 \times 3, 32 \end{bmatrix} \times 2$$
Transition 3	$15 \times 11$	$1 \times 1$, 512
	$7 \times 5$	Average pool
Dense block 4	$7 \times 5$	$$\begin{bmatrix} 1 \times 1, 128 \\ 3 \times 3, 32 \end{bmatrix} \times 1$$
Classification layer	$1 \times 1$	Adaptive average pool
		Fully connected, softmax

We apply two recent architectures in our analysis called CovidResNet and CovidDenseNet [36]. They were recently proposed to detect COVID manifestations in CT scan images and have proven to generalize well on small-scale CT datasets with a few thousand images. CovidResNet and CovidDenseNet have a total of 4.98 million parameters and 1.63 million parameters, respectively. Tables 3 and 4 give detailed information about the CovidResNet and CovidDenseNet architectures [36], their layers and feature map sizes. In both architectures the first convolution operation has a $7 \times 7$ kernel and a stride of 2. Subsequently, a max pooling operation further reduces the size of the feature maps.

The architecture of CovidResNet continues with a sequence of residual blocks [33]. to get the vanishing gradient problem and the performance degradation problem under control [33]. Each basic block consists of a skip connection, two $3 \times 3$ convolutions and a summing operation as can be seen in Fig. 4 on the left-hand side. One residual blocks is shown with the input layer on the left-hand side. The computational graph splits into two different branches, the closer one being the skip connection. The other branch contains two $3 \times 3$ convolution operations. The resulting feature maps are shown in the visualization as separate layers. Finally, the convolution output and the skip connection output are summed up, which can be seen in the final layer in the image.

CovidDenseNet [36] uses dense blocks [37] instead of residual blocks. A dense block is illustrated in Fig. 4 on the right-hand side. The input layer is located on the bottom left in the image. The input is convoluted once and the result is concatenated with the input. This procedure is repeated a couple of times, such that the total number of feature maps increases. The output of the dense block is shown at the very top of the image.

## Results and discussion

### Model performance

**Table 5:** Performance metrics for the three different training strategies

Architecture	Dataset	Type	Test accuracy
CovidResNet	Caltech	Standard	0.78
CovidResNet	Caltech	Shuffled labels	0.01
CovidResNet	Caltech	Noisy samples	0.42
CovidResNet	SARS-CoV-2	Standard	0.96
CovidResNet	SARS-CoV-2	Shuffled labels	0.49
CovidResNet	SARS-CoV-2	Noisy samples	0.97
CovidDenseNet	SARS-CoV-2	Standard	0.97
CovidDenseNet	SARS-CoV-2	Shuffled labels	0.50
CovidDenseNet	SARS-CoV-2	Noisy samples	0.99

Table 5 summarizes the performance metrics of the trained models. At the end of the training process, the models have zero error on the train set. The results show that the three training strategies lead to models with different levels of generalization abilities.

The first training strategy on Caltech101 with CovidResNet achieves a test accuracy of $78\%$. CovidResNet and CovidDenseNet reach test accuracies of $96\%$ and $97\%$ on the SARS-CoV-2 dataset, respectively.

The second learning strategy with shuffled training labels performs as good as a random classifier with $1\%$ test accuracy on Caltech101 and about $50\%$ on SARS-CoV-2, which is also in line with our expectations.

Our third strategy with noisy samples reached a test accuracy of only $42\%$ on Caltech101. It generalizes worse than the first network, but its ability to classify the test data correctly in almost every second case considering 101 classes is remarkable. We find it surprisingly difficult to get a network, which has a zero training error, but generalizes badly, proving the good bias of CovidResNet and CovidDenseNet for image recognition problems, as well as CNNs in general. On SARS-CoV-2 the test accuracy of the CovidResNet and CovidDenseNet is similar to the first training approach. One should notice, that these two models managed to perform two leanings at once: The memorization of many train images and the development of generic features. We visualize the networks to learn how memorization and generalization can coexist within CNNs in the subsequent section.

### Memorization correlates with the processing of local information

**Fig. 7:**
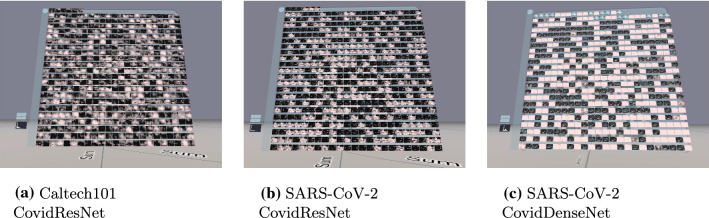
First training strategy: activations of the last convolutional layer

**Fig. 8:**
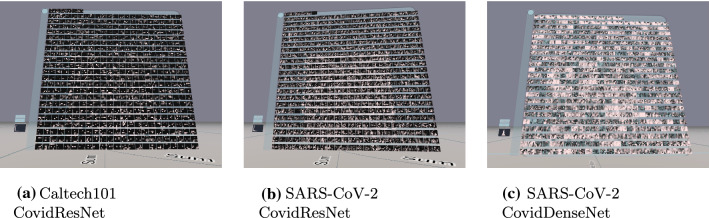
Second training strategy: activations of the last convolutional layer. Before training, all labels in the train set were shuffled

**Fig. 9:**
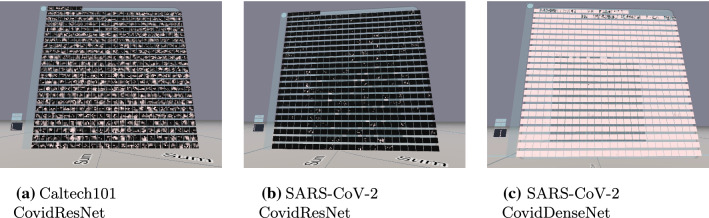
Third training strategy: activations of the last convolutional layer. The train set was copied 9 times and each copy received white noise and random labels. The original dataset with original labels is contained exactly once

This section compares activation patterns within networks with different generalization abilities. Figures 7, 8 and 9 show the activations of the last convolutional layer for the three training approaches and different architectures. The input image is a sunflower or a COVID CT image, respectively. The white color denotes high activation, while blue means negative values and black zero.

#### First training strategy

Figure 7 presents the 512 feature maps of the last convolutional layer of CovidResNet and CovidDenseNet trained with the first training strategy. Active channels spread their activity over large spatial areas and neighboring pixels tend to have a similar level of activation. The channels tend to be either activated or not. We observed the activations of many input images and found empirically no test image with significantly different findings.

#### Second training strategy

Figure 8 shows the activation in networks trained with shuffled train labels and no generalization abilities. The feature maps contain a lot of spatial information. Almost all channels show some level of activity. The 512 image filters seem to respond to various local image regions spreading over the entire image space. There is no hint that the channels process any semantic information or mark consistent image regions, where the sunflower or Covid manifestations are located. We assume that very specific textures or even pixel-to-pixel correlations are processed in the last layer. We will support this assumption later using feature visualization.

#### Third training strategy

The feature maps in Fig. 9 contain plenty of local information. The neighbors of active pixels do not necessarily have similar levels of activation, which suggests the processing of high-frequency patterns. This characteristic applies for all three networks trained with the third training strategy. However, CovidResNet trained on the SARS-CoV-2 dataset has sparser activations compared to the Caltech101 model. CovidDenseNet has a similar behavior with flipped sign. It is a hint, that some channels dominate others. Possibly they strongly fire when confronted with image-specific properties letting the network identify the train images.

### Feature visualization on the caltech101 dataset

**Table 6:**
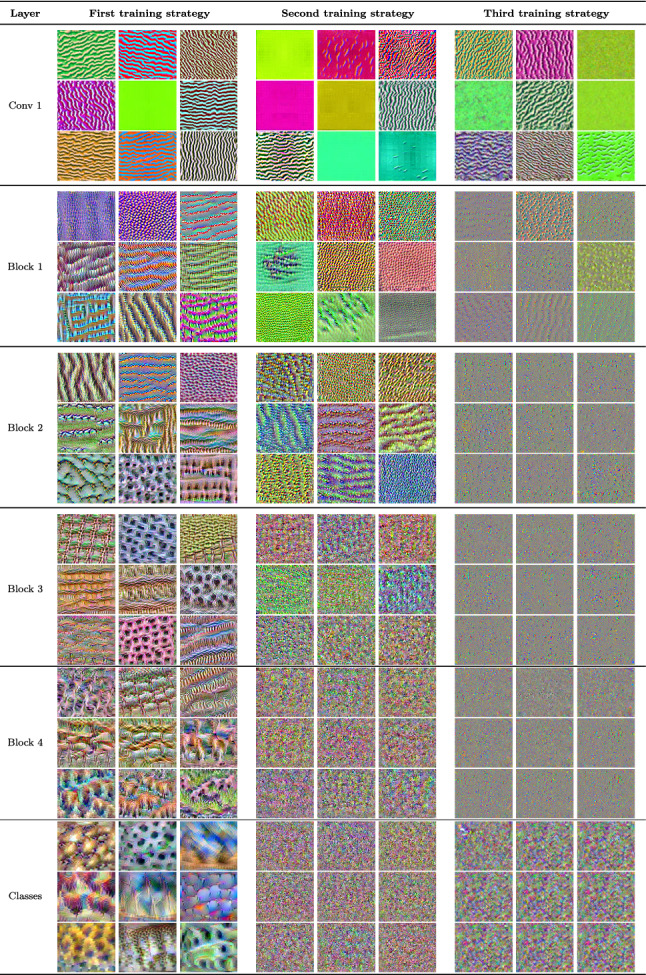
Feature visualization for single channels in CovidResNet trained on the Caltech101 dataset with different training strategies. Best viewed in color with zoom

We apply feature visualization to analyze how the CNNs are able to both memorize images and keep the ability to generalize at the same time. Table 6 shows the results for feature visualization applied on CovidResNet trained on the Caltech101 dataset. We strongly recommend to view the visualizations digitally and to zoom into the images to see the great level of detail and high-frequency patterns.

#### First training strategy

The first column in Table 6 consists of images, which maximize the activation of specific channels in the network that was trained with the first training strategy. Apparently, the filters in the first convolutional layer have learnt edge filters in various orientations with different color preferences. Some channels focus more on color than structure. The image filters in block 1 are susceptible to monotonous textures, some containing small-scale, some larger structures. Stripes and grid-like patterns from the first layer are still visible, indicating the hierarchical filter structure of residual networks. The feature visualizations in the second block of CovidResNet look like more complex versions of the previous block with more detail. The two last images of the third row seem to show copies of shapes, which emerged from a grid-like pattern and a horizontal bar pattern. The complexity of the textures increase in blocks 3 and 4, where first instances of objects or animals can be observed. One image could show trees standing next to each other, which seem to have originated from horizontal stripe patterns. Some of the images may show birds, some mammals and sometimes undefinable objects, which is a strong hint for the processing of semantics in this layer.

The feature visualization of the classification neurons unveils what visual concepts the CNN has learned from the classes. Please see Fig. 6 for references from the dataset. The first image is supposed to be a crocodile head and shows two phenomena, typically found on a crocodile head: Large scales and eyes. The arrangement of these elements seems to be irrelevant to the network. The second image shows what the network thinks to be a panda. The reader may notice the large, black spots, which are characteristic for panda faces. The third image is supposed to be a pyramid. Usually, pyramids stand on a sandy ground and the sky is blue. Triangular shapes populate the image. The first image of the second row shows a group of roosters, the second a schooner and the third the cartoon character Snoopy. Remarkably, Snoopy is detected by the characteristic form of his snout and the small, black dog nose. The last row illustrates a sunflower, a wild cat and a Yin Yang symbol. For the cat the network simply detects dotted fur patterns and a bright belly. No body parts or heads are visible. The arrangement of Ying and Yang shapes are also irrelevant.

#### Second training strategy

In the first layer the feature visualizations show edges and repetitive noise patterns. The high-frequency patterns are only visible using zoom. In block one and two the feature visualizations are much noisier and less plastic than for the first training strategy. The channels do not focus on structure or shape but on combinations of colorful pixels. The deeper layers are dominated by specific high-frequency patterns and pixel combinations. There is no hint, that the CNN processes any semantic information.

#### Third training strategy

The feature visualization of CovidResNet trained with the third training strategy share much similarity to the second training strategy. However, in the first block, the patterns deviate strongly from the first two models because the visualizations are dominated by distinct groups of pixels. In the subsequent layers, all large structures disappear. The visualization of the classification neurons show rough, high-frequency patterns without any global structure.

### Feature visualization on the SARS-CoV-2 dataset

The results of the feature visualization technique for CovidResNet and CovidDenseNet trained on the SARS-CoV-2 dataset are shown in Tables 7 and 8, respectively. As the feature visualization of the architectures are similar to each other, they are discussed jointly.

**Table 7:**
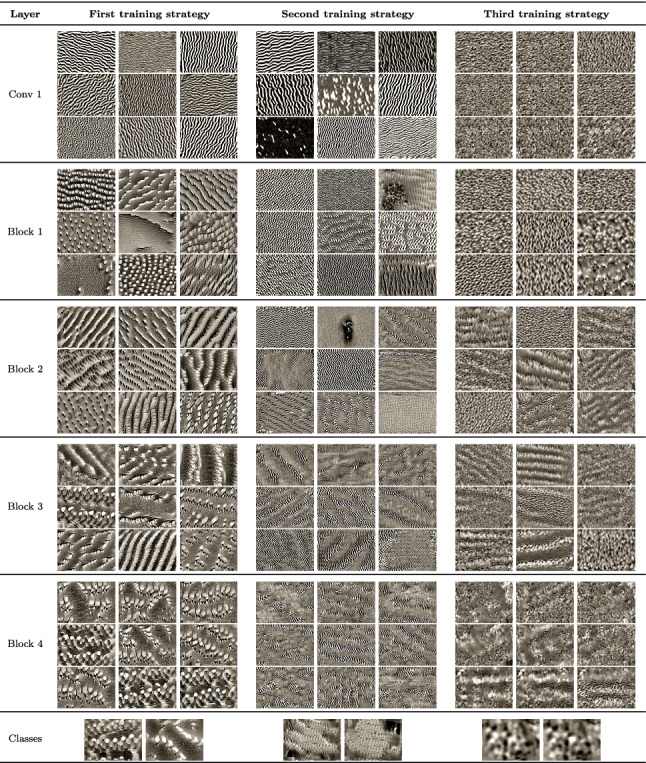
Feature visualization for single channels in CovidResNet trained on the SARS-CoV-2 CT-scan dataset with different training strategies. Best viewed in color with zoom

**Table 8:**
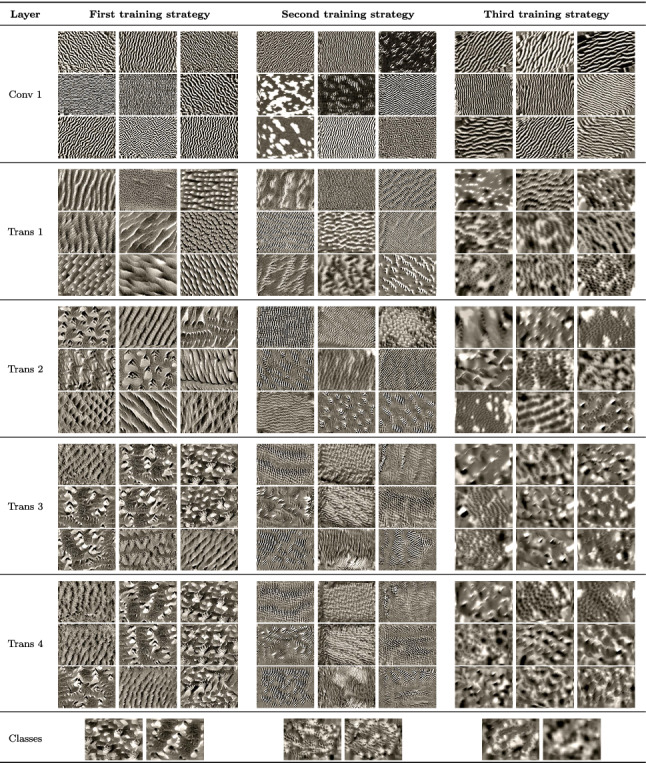
Feature visualization for single channels in CovidDenseNet trained on the SARS-CoV-2 CT-scan dataset with different training strategies. Best viewed in color with zoom

#### First training strategy

The filters in the first layer of the CNNs trained with the first training strategy are susceptible to edges and grid-like patterns. Deeper in the network, the stripes and grid patterns evolve into characteristic shapes with different levels of roughness and smoothness. In contrast to Caltech101, the SARS-CoV-2 models loose diversity in the deeper layers. We see that the channels in the fourth layer can be roughly clustered into two groups, one obviously related to COVID cases and one to Non-COVID cases. The feature visualization of the classification layer reveals manifestations of the COVID disease.

#### Second training strategy

Similar to the models trained on the Caltech101 dataset, the layers are susceptible to local features, tiny edges and riffles in different orientations. A high density of high-frequency patterns exists in the generated images, which can only be seen with zoom enabled. There is no hint for the processing of semantic information.

#### Third training strategy

The models trained with the third training approach had to memorize many images and are also able to generalize. Large structures in the feature visualizations exist, but are not as defined as in the first training strategy. This is a hint, that semantic relationships are processed by the network, but there are also high-frequency patterns. The good generalization abilities of the models imply that the class neurons must be susceptible to both, Covid manifestations and memorized noise patterns. It appears, that the formation of Covid manifestations does not occur, because the noise patterns, which are used to memorize the images, already maximize the activation. The feature visualization algorithm might be biased toward finding high-frequency patterns. Thus, there is no incentive to generate any objects or textures related to Covid infections. We assume that the networks contain subnetworks, which are susceptible to the memorized noise patterns. These subnetworks dominate the feature visualization, such that the formation of Covid manifestations does not occur and the optimization gets stuck in a local minimum.

### Discussion

The visualization suggests that the memorization of images involves the processing of high-frequency patterns and the memorization of local pixel arrangements. Interestingly, it is possible for the same CNN to learn both, generic features and high-frequency patterns. These two modalities are not mutually exclusive and they are entangled. No channels could be identified, that process exactly one modality. Possibly, memorization happens in entangled subnetworks. Triggering the high-frequency memorization subnetwork could be a lever for adversarial attacks, where a slight variation in the input image changes the decision of the network drastically, motivating the development of new architectures and training algorithms with lower risk of memorizing images.

In order to discourage neural networks from memorizing images, one approach is to remove high-frequency patterns from the training images, such that it is impossible track down specific pixel arrangements. However, blurring input images can remove relevant information needed for classification. Also, the absence of high frequencies might make the trained network less robust when confronted with real-world scenarios. Another idea is to augment the training data with noise, which is a standard approach to make networks robust toward high-frequency image data. Another idea is the suppression of local information processed in the network by adding random noise to the activations. However, there is the risk of negatively affecting the performance and it is unclear how to choose the trade-off between noise and lowering the training loss. Some regularization strategies prune information from the network internals, for instance dropout. Moreover, one could introduce a total variation loss on the feature maps. An easier implementation could be Gaussian blurring on layer activations to destroy some of the spatial information.

The generalization ability of CNNs could benefit from a simplification of the visual concepts processed in the layers. The analysis of the well-generalizing neural networks showed the immense complexity and variability of features within the deeper layers. One form of simplification is the reduction in model parameters, for example by the use of depth wise convolution. As a next step one could try to make the $3 \times 3$ convolutions of the depth wise convolution handcrafted to further reduce the number of parameters and simplify the patterns a CNN is susceptible to. This idea breaks with the paradigm that CNNs should learn all of their convolutional filters. With an appropriate set of filter kernels the CNN would only learn how to combine and weight the pre-defined filters using linear combinations via trainable $1 \times 1$ convolutions.

## Conclusions

This work presents open-source software for the immersive visualization of popular CNN architectures using Python and the Unity game engine, where the user can freely walk in a 3D environment in VR or desktop mode. The user can move, turn around and change the perspective, which gives a good overview of complex architectures. Feature maps, activation histograms, weight histograms and feature visualizations provide information to improve the design of layers and the architecture, encourage exploration and lead to a deeper understanding of CNNs. We think that the 3D environment promotes a deeper understanding of deep networks, because much more information can be arranged in 3D compared to 2D while keeping clarity and tidiness.

We addressed the problem of how to make the visualization of large-scale models feasible in VR. Therefore, we developed a Pytorch module to allow the optimized visualization of almost arbitrary computational graphs in Unity including branches and joints. In the client-server approach the Python server handles the network architecture using Pytorch. The ability to quickly visualize large-scale networks in 3D will probably make immersive approaches more relevant to the deep learning community. The software targets developers and researchers, as well as newcomers to deep learning.

In a use case study, we analyzed how CNNs memorize images. We trained the architectures CovidResNet and CovidDenseNet on the Caltech101 and the SARS-CoV-2 datasets using three different training strategies to get models with different generalization abilities. For getting bad generalizing models we proposed a new training method where we trained with multiple copies of the training data, random labels and some additive white noise to make the copies distinguishable. Using the visualization software we concluded that the CNNs memorized images based on high-frequency patterns. It appears that CNNs can contain subnetworks, that are susceptible to specific local pixel arrangements. These findings nourish the threat of adversarial attacks. The use case study motivates the usage of regularization techniques like dropout, blurring of feature maps or adding random noise to them. We also suggest new measures to make it harder for the network to memorize images involving the combination of deep learning with handcrafted filter kernels.

The ability to visualize popular, state-of-the-art architectures raises new questions for future work. With the visualization embedded in a 3D world space it seems convenient to consider machine learning problems with 3D input data such as in pose estimation [38, 39]. Applying a 3D visual analysis system would be interesting, because not only the networks, but also the input data can be shown in 3D. Another point for future work is the visualization of statistical variations in network features. The visualization could be extended to make these variations accessible to the user. Also, one could combine feature maps using PCA [40, 41]. Another idea is to cluster the channels according to the cross-correlation of their filter outputs. This could further improve the presentation of the feature space and illustrate the semantic connections between different channels.

## Data Availability

The Caltech101 [34] and the SARS-CoV-2 [35] datasets that are analyzed during this study are openly available from the public data platform Kaggle at www.kaggle.com/datasets/athota1/caltech101 and www.kaggle.com/datasets/plameneduardo/sarscov2-ctscan-dataset, respectively.
